# Supplementary material for: Global stabilization of the transcriptome in mitotic cells
Source: EMBO J. 2026 Apr 9;45(10):3563–88. doi: 10.1038/s44318-026-00765-5 (PMC13187299; doi:10.1038/s44318-026-00765-5)
Supplement: Supplementary file 1 — Table EV1 [file 44318_2026_765_MOESM1_ESM.docx]

**Global stabilization of the transcriptome in mitotic cells**

**Expanded View Materials**

**Table EV1.** siRNA sequences

| **siRNA** | **Sequence** |  |  |  |
| --- | --- | --- | --- | --- |
| siNC (cocktail) | UAGGACUAAACACAUCAA | UAAGGCUAUGAAGAGAUAC | AUGUAUUGGCCUGUAUUAG | AUGAACGUGAAUUGCUCAA |
| siGFP | GAACGGCAUCAAGGUGAACUU | |  |  |
