# Supplementary material for: Global stabilization of the transcriptome in mitotic cells
Source: EMBO J. 2026 Apr 9;45(10):3563–88. doi: 10.1038/s44318-026-00765-5 (PMC13187299; doi:10.1038/s44318-026-00765-5)
Supplement: Supplementary file 2 — Table EV2 [file 44318_2026_765_MOESM2_ESM.docx]

**Global stabilization of the transcriptome in mitotic cells**

**Expanded View Materials**

**Table EV2.** RT-qPCR primers

| **Target** | **Sequence** |
| --- | --- |
| Nluc Forward | TGTACCCTGTGGATGATCATCAC |
| Nluc Reverse | ATTTTGTTGCCGTTCCACAG |
| GAPDH Forward | TCGGAGTCAACGGATTTGGT |
| GAPDH Reverse | TTCCCGTTCTCAGCCTTGAC |
| GFP 3' Forward | CCGACCACTACCAGCAGAAC |
| GFP 3' Reverse | CGCTTCTCGTTGGGGTCTTT |
| GFP 5' Forward | GGACGACGGCAACTACAAGA |
| GFP 5' Reverse | AAGTCGATGCCCTTCAGCTC |
