# Supplementary figures and images for: Global stabilization of the transcriptome in mitotic cells
Source: EMBO J. 2026 Apr 9;45(10):3563–88. doi: 10.1038/s44318-026-00765-5 (PMC13187299; doi:10.1038/s44318-026-00765-5)

DMSO

IAA

0h

1h

4h

0h

1h

4h

PABPC1

alpha-tubulin

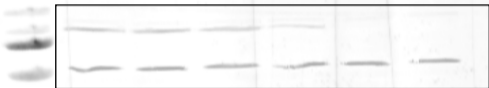

Supplement: Supplementary file 8 — Source data Fig. 7 [file 44318_2026_765_MOESM8_ESM.zip › Figure 7/7A/PABPC1_tubulin.pdf]

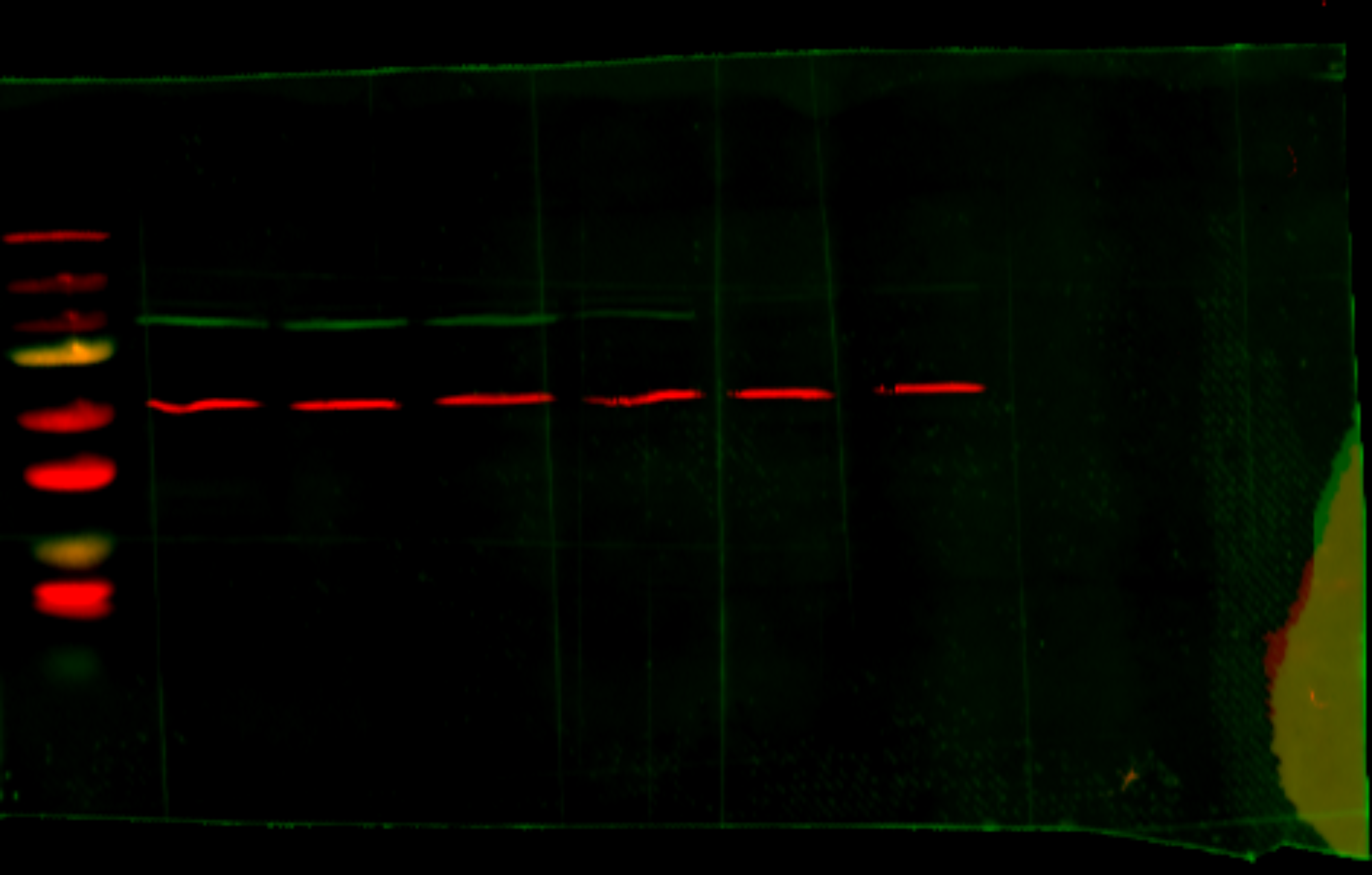

Supplement: Supplementary file 8 — Source data Fig. 7 [file 44318_2026_765_MOESM8_ESM.zip › Figure 7/7A/20241213_KK_PABPC-AID_STLC.tif]
