# Supplementary material for: Global stabilization of the transcriptome in mitotic cells
Source: EMBO J. 2026 Apr 9;45(10):3563–88. doi: 10.1038/s44318-026-00765-5 (PMC13187299; doi:10.1038/s44318-026-00765-5)
Supplement: Supplementary file 9 — Figure EV5 Source Data [file 44318_2026_765_MOESM9_ESM.zip › Figure EV5/EV5A/PABPC1_PABPC4_actin_vinculin.pdf]

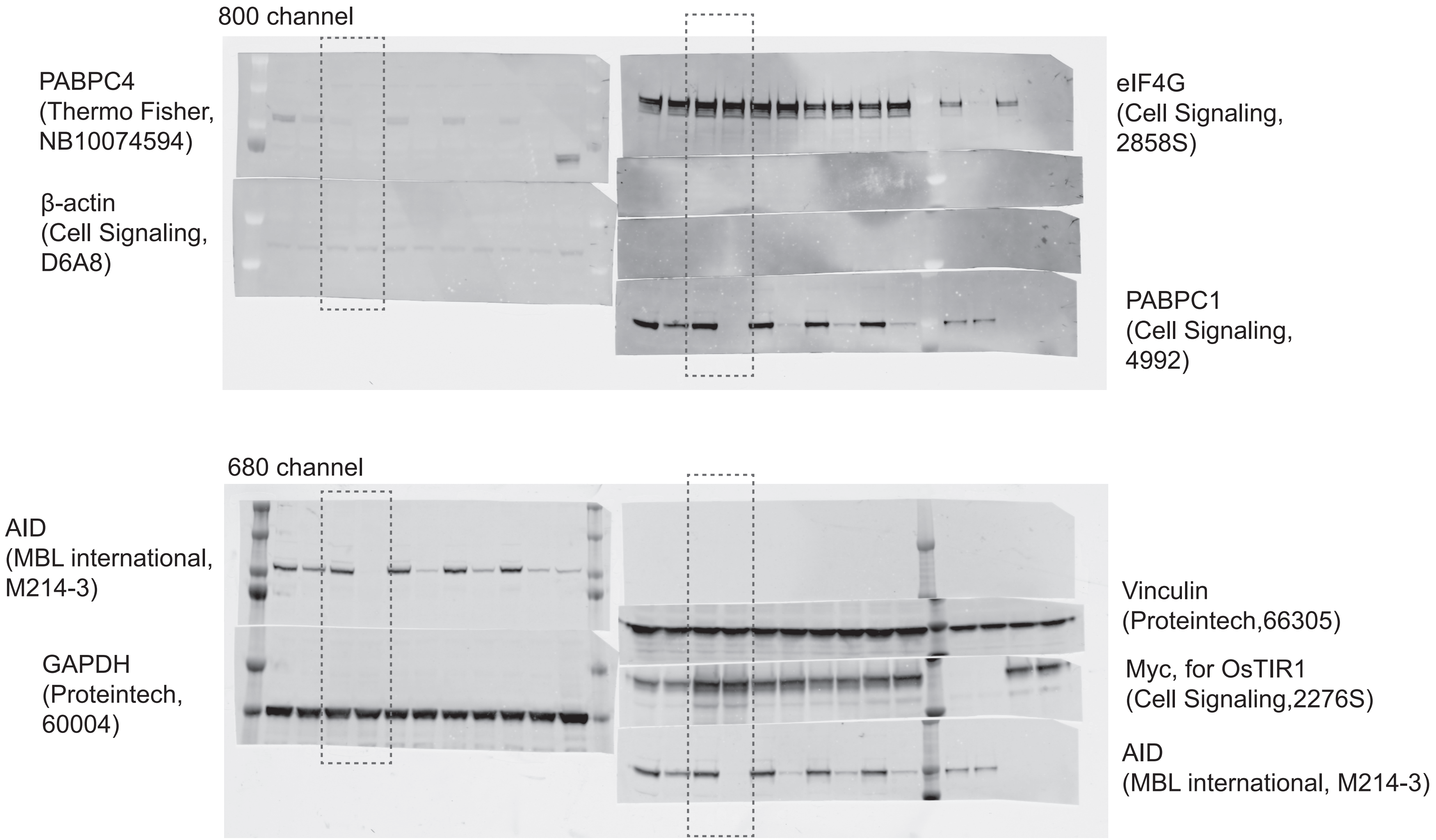
800 channel
PABPC4
(Thermo Fisher,
NB10074594)
β-actin
(Cell Signaling,
D6A8)
eIF4G
(Cell Signaling,
2858S)
PABPC1
(Cell Signaling,
4992)
680 channel
AID
(MBL international,
M214-3)
GAPDH
(Proteintech,
60004)
Vinculin
(Proteintech,66305)
Myc, for OsTIR1
(Cell Signaling,2276S)
AID
(MBL international, M214-3)
