# Supplementary material for: Global stabilization of the transcriptome in mitotic cells
Source: EMBO J. 2026 Apr 9;45(10):3563–88. doi: 10.1038/s44318-026-00765-5 (PMC13187299; doi:10.1038/s44318-026-00765-5)
Supplement: Supplementary file 10 — Expanded View Figures [file 44318_2026_765_MOESM10_ESM.pdf]

Expanded View Figures

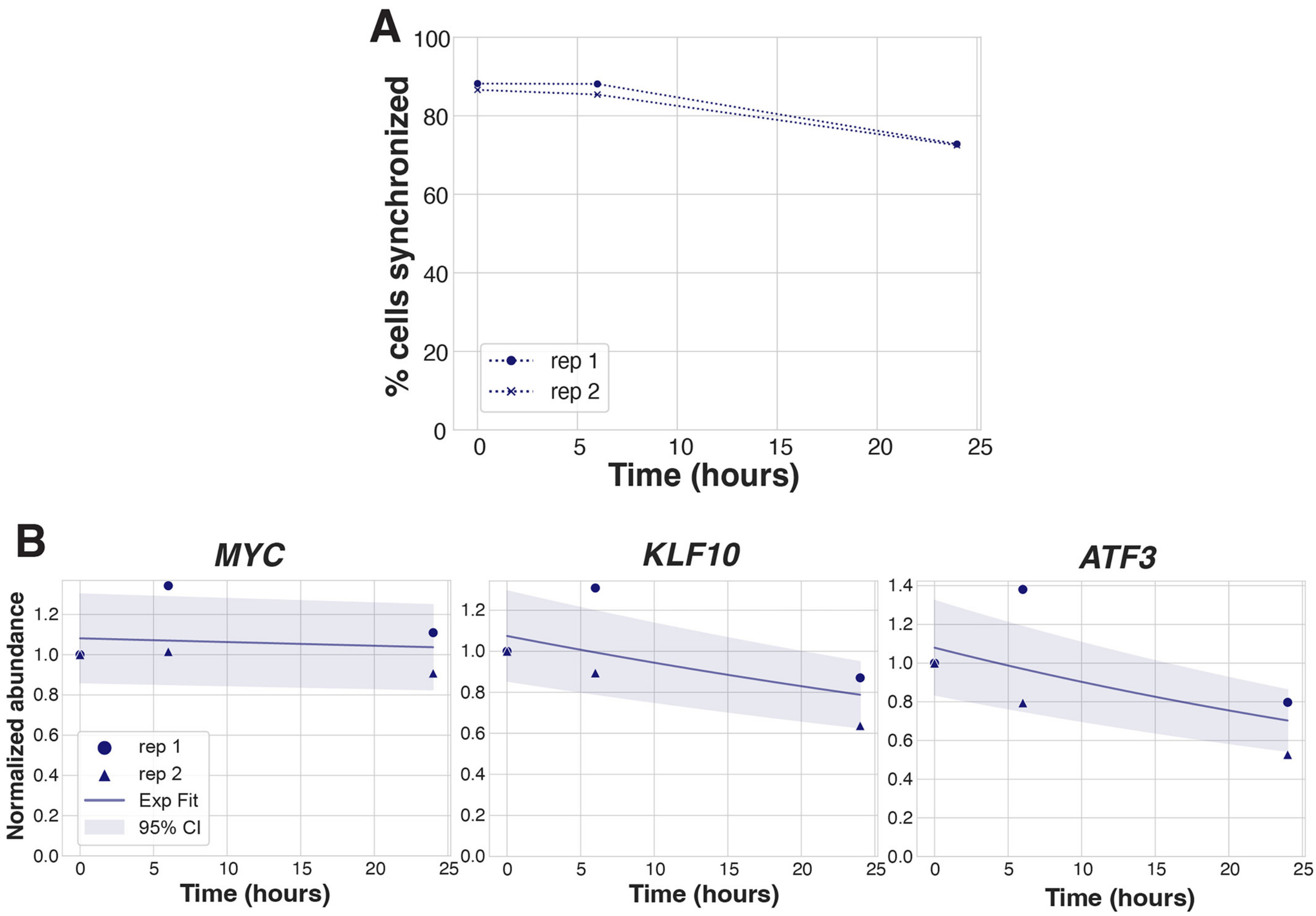

Figure EV1. Synchronization efficiency and transcript abundances during prolonged mitotic arrest.

(**A**) Percentage of cells synchronized in mitosis during each experimental timepoint (Fig. 1), as quantified by FACS analysis of DNA content and pH3(Ser10). (**B**) Exponential curve fit to individual transcript abundances in STLC-arrested cells, plotted with 95% confidence intervals. *MYC* $t_{1/2}$ 399 h, R^2 0.05. *KLF10* $t_{1/2}$ 54 h, R^2 0.72. *ATF3* $t_{1/2}$ 39 h, R^2 0.78.

 © The Author(s)

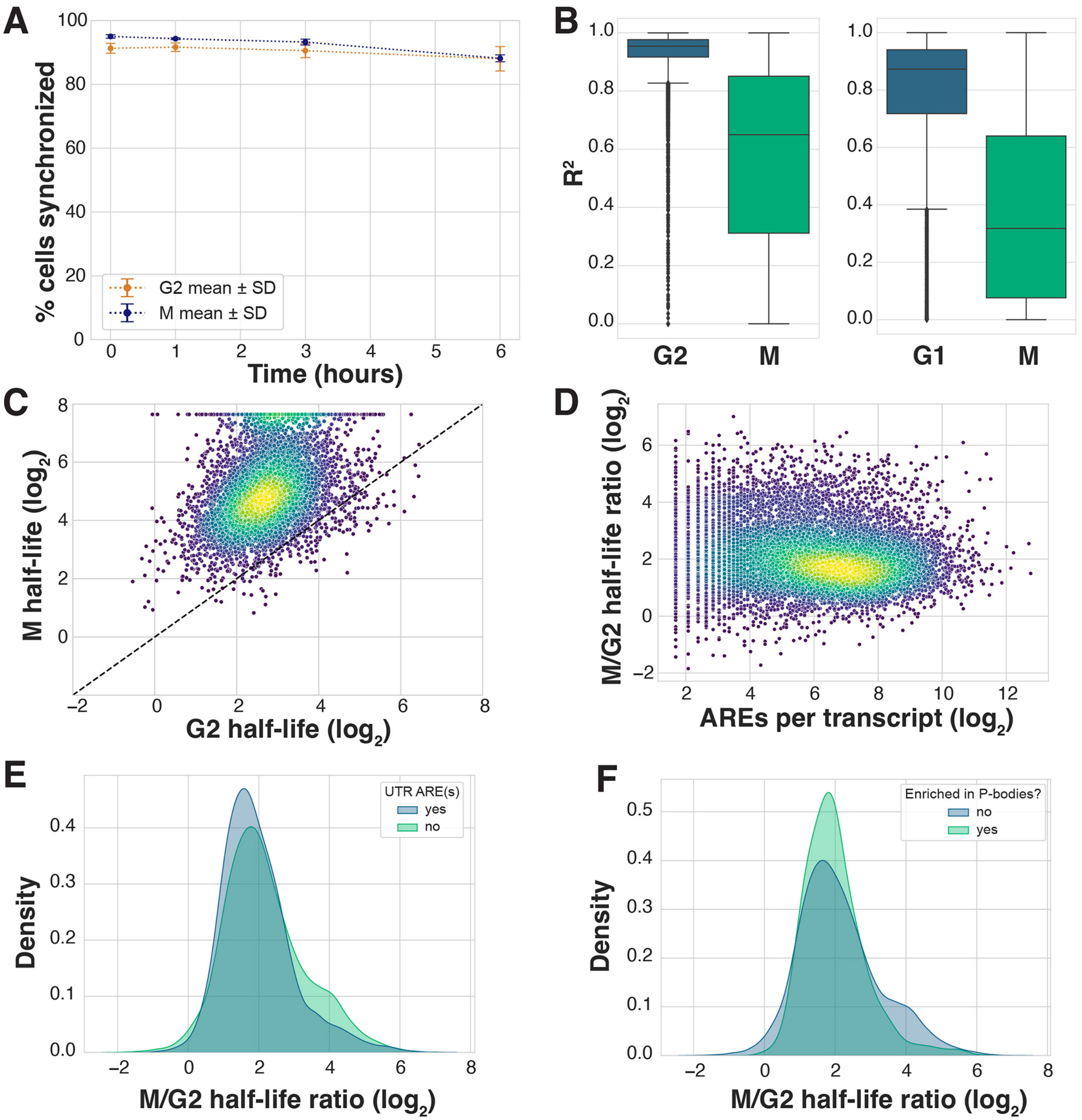
A
% cells synchronized
Time (hours)
G2 mean ± SD
M mean ± SD
B
R²
G2
M
G1
M
C
M half-life (log2)
G2 half-life (log2)
D
M/G2 half-life ratio (log2)
AREs per transcript (log2)
E
Density
UTR ARE(s)
yes
no
M/G2 half-life ratio (log2)
F
Density
Enriched in P-bodies?
no
yes
M/G2 half-life ratio (log2)

Figure EV2. Analyses related to transcription inhibition timecourses.

(**A**) Percentage of cells synchronized in G2 or mitosis during each experimental timepoint (Fig. 2), as quantified by FACS analysis of DNA content and pH3(Ser10). (**B**) Boxplots showing R^2 goodness of fit values for half-life calculations for transcripts mapped in transcription inhibition experiments (Fig. 2B, C). Each box shows the interquartile range (IQR) of the data, with the median marked by a horizontal line. The whiskers extend to include the rest of the distribution, except for points that are determined to be outliers based on the IQR. R^2 cutoffs were chosen based on interphase fits in order to allow for the possibility of global mRNA stabilization, as a lack of degradation is expected to result in poorer fit values. $n = 10{,}544$ (left), $n = 10{,}604$ (right). (**C**) Scatterplot comparing mRNA half-lives in cells synchronized in G2 or M and treated with THZ1. Data from one biological replicate. R^2 cutoff >0.4 was used, $n = 6410$. Median $t_{1/2}$ 6.77 and 33.44 h for G2 and M, respectively. (**D**) Scatterplot comparing mitotic stabilization and number of AREs per transcript. ARE annotations were acquired from AREsite2 (Fallmann et al, 2016). Arbitrary $c = 0.9$ was added to all ARE counts to allow log-transformation of the data. $R_S = -0.13$. (**E**) Probability density function plot comparing the degree of mitotic stabilization among transcripts with or without AREs in the 3′ UTR. 3′ UTR ARE annotations were acquired from https://brp.kfshrc.edu.sa/ared/Home/FTP (Bakheet et al, 2018). $n = 3085$ transcripts with AREs in 3′ UTR, $n = 6773$ transcripts with no AREs in the 3′ UTR. (**F**) Probability density function plot comparing the degree of mitotic stabilization among transcripts based on their P-body enrichment status (Hubstenberger et al, 2017). P-body enrichment was defined by FDR <0.01 and $\log_2$ enrichment >2, resulting in 1602 P-body-enriched transcripts and 8256 not enriched.

 © The Author(s)

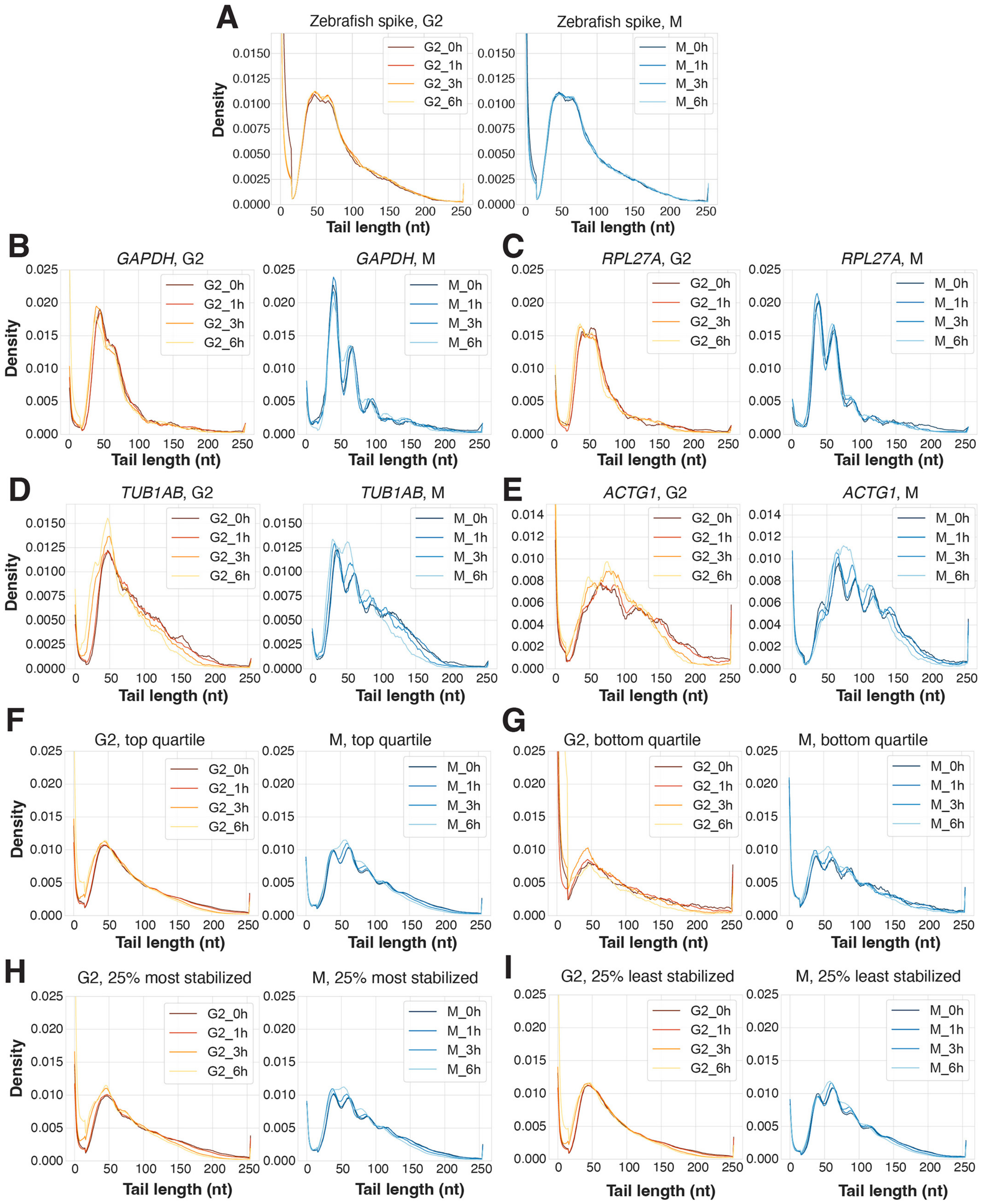
A
Zebrafish spike, G2
Zebrafish spike, M
Density
Tail length (nt)
G2_0h
G2_1h
G2_3h
G2_6h
M_0h
M_1h
M_3h
M_6h
B
GAPDH, G2
GAPDH, M
C
RPL27A, G2
RPL27A, M
D
TUB1AB, G2
TUB1AB, M
E
ACTG1, G2
ACTG1, M
F
G2, top quartile
M, top quartile
G
G2, bottom quartile
M, bottom quartile
H
G2, 25% most stabilized
M, 25% most stabilized
I
G2, 25% least stabilized
M, 25% least stabilized

◀ **Figure EV3. Poly(A) tail-length distributions for subsets of transcripts in G2- and M-arrested cells.**

(**A**) Poly(A) tail-length distributions from zebrafish mRNA spiked into G2- or M-treated samples as measured by PAL-seq. (**B–E**) Poly(A) tail-length distributions in G2- or M-arrested cells treated with actinomycin D as measured by PAL-seq for GAPDH (**B**), RPL27A (**C**), TUB1AB (**D**), ACTG1 (**E**). (**F**, **G**) Poly(A) tail-length distributions in G2- or M-arrested cells treated with actinomycin D as measured by PAL-seq for 25% most highly expressed genes (**F**) or 25% mostly lowly expressed genes (**G**). (**H**, **I**) Poly(A) tail-length distributions in G2- or M-arrested cells treated with actinomycin D as measured by PAL-seq for 25% most stabilized transcripts (**H**) or 25% least stabilized transcripts (**I**) based on analysis from Fig. 2.

 © The Author(s)

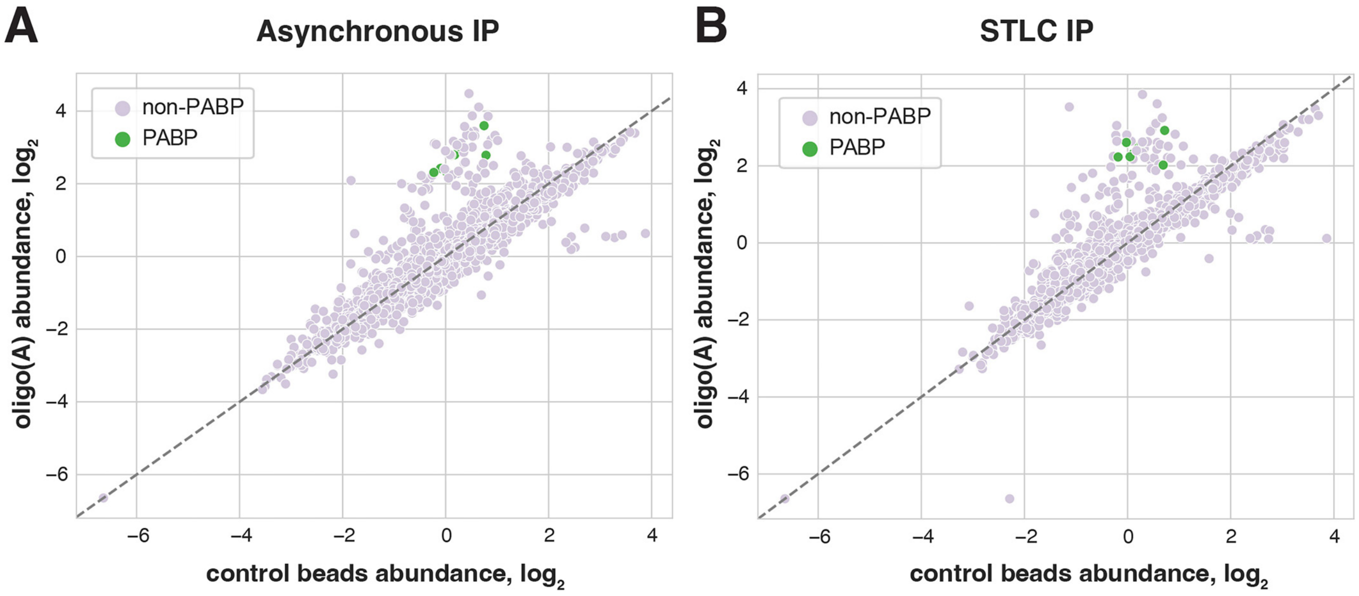

Figure EV4. Protein abundances in control and oligo(A) pulldowns in asynchronous and STLC-arrested cells.

(**A**, **B**) Scatterplots comparing protein abundance in pulldowns by oligo(A)-coated beads and negative control beads in (**A**) asynchronous and (**B**) STLC-arrested cells. Poly(A)-binding proteins are highlighted in green.

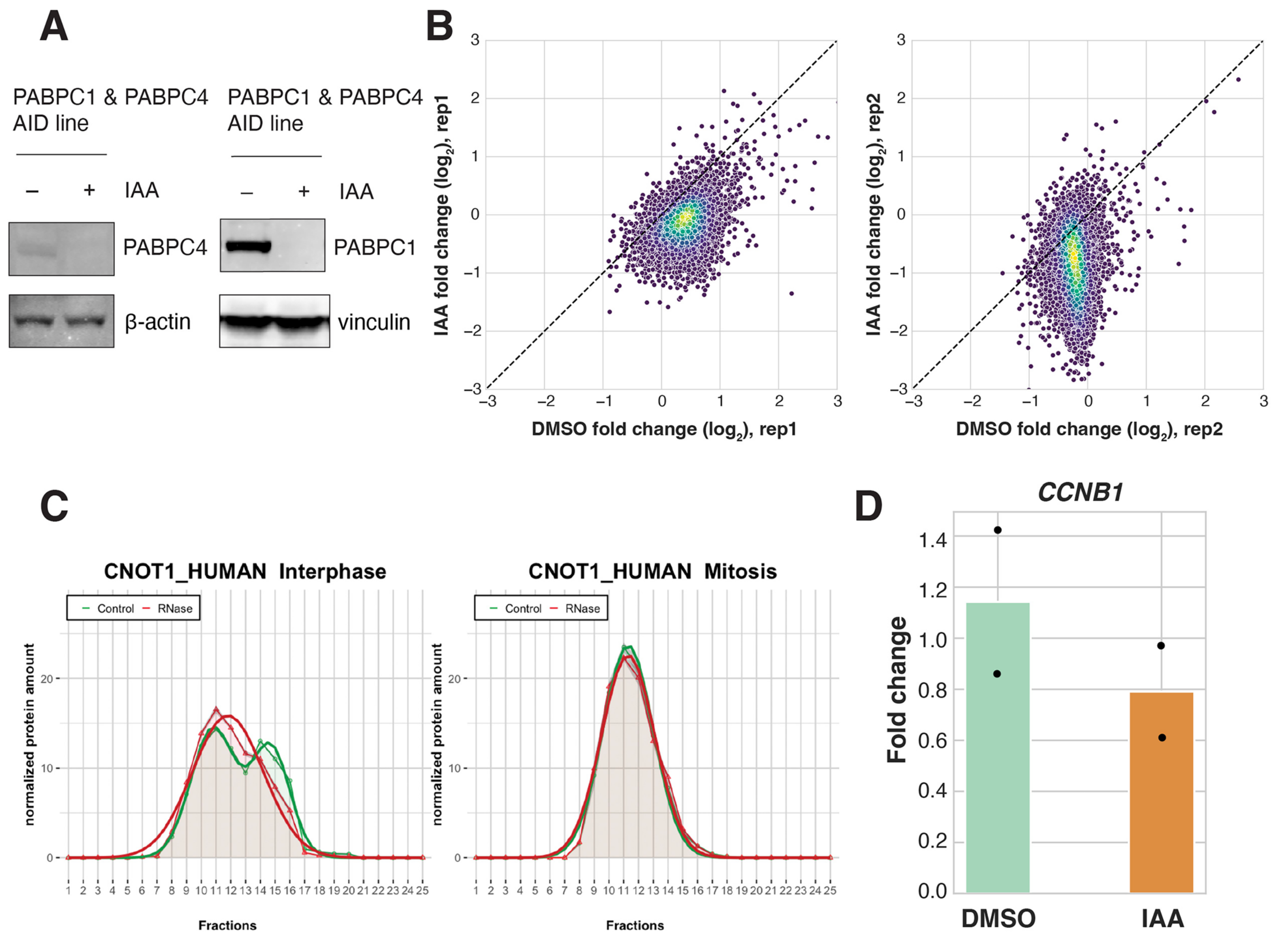

Figure EV5. Data related to PABPC1&4 depletion experiments.

(**A**) Western blots showing PABPC1 & PABPC4 depletion efficiency after 1 h of IAA treatment. Beta-actin and vinculin are shown as loading controls. (**B**) Scatterplots comparing mRNA abundance fold changes in STLC-arrested HCT116 cells within 4 h of either DMSO control treatment or IAA-induced PABPC1&4 depletion. Replicates 1 & 2, $n = 11{,}078$. (**C**) Graphical representation of the CNOT1 protein amount from interphase or mitotic HeLa cells in 25 different fractions of control (green) and RNase-treated (red) sucrose density gradients analyzed by mass spectrometry. Plots were acquired from https://r-deep3.dkfz.de/ (Rajagopal et al, 2025). The leftward shift of the distribution upon RNase treatment in interphase cells suggests interaction with RNA, whereas a lack of shift in mitotic cells suggests reduced or no interaction. (**D**) Bar graph comparing *CCNB1* degradation in STLC-arrested HCT116 cells within 4 h of either DMSO control treatment or IAA-induced PABPC1&4 depletion. The mean of two biological replicates is plotted; each point represents one biological replicate. Source data are available online for this figure.

 © The Author(s)
